# Production system-associated hepatic histomorphometric adaptations in swine: Comparative analysis of glycogen deposition, Kupffer cell abundance, and liver microarchitecture

**DOI:** 10.14202/vetworld.2026.3295-3310

**Published:** 2026-08-04

**Authors:** Nattawat Chaiyawong, Anongwadee Nadprasop, Natcha Khong-on, Siwakon Kongchan, Ittipon Phoungpetchara, Pichaya Jumnongprakhon, Charkriya Promsuban

**Affiliations:** 1 Siriraj Integrative Center for Neglected Parasitic Diseases, Department of Parasitology, Faculty of Medicine Siriraj Hospital, Mahidol University, Bangkok 10700, Thailand.; 2 Department of Anatomy, Faculty of Medical Science, Naresuan University, Phitsanulok 65000, Thailand.; 3 Center of Excellence in Medical Biotechnology, Faculty of Medical Science, Naresuan University, Phitsanulok 65000, Thailand.

**Keywords:** beta-agonist-free production, glycogen deposition, hepatic microarchitecture, histomorphometry, Kupffer cells, periodic acid–Schiff staining, swine, sustainable livestock production

## Abstract

**Background and Aim:**

Swine production systems differ considerably in housing, nutrition, biosecurity, and environmental exposure, all of which may influence hepatic metabolism, immune function, and tissue architecture. Histological indicators such as hepatic glycogen deposition, Kupffer cell abundance, and sinusoid–hepatic cord organization provide valuable insight into physiological adaptations associated with different management practices. However, comparative information on these hepatic characteristics across commercial swine production systems remains limited. This study aimed to compare hepatic glycogen accumulation, Kupffer cell abundance, and hepatic microarchitecture among clinically healthy swine raised under beta-agonist-free, hygienic, and natural production systems in Thailand.

**Materials and Methods:**

A cross-sectional comparative histomorphometric study was conducted using liver tissues collected from 30 clinically healthy market weight commercial crossbred swine (10 animals per production system). Liver samples were fixed in neutral buffered formalin, processed routinely, and stained using the periodic acid–Schiff method. Histomorphometric analyses included quantification of periodic acid–Schiff-positive glycogen area, zone-specific glycogen distribution within hepatic lobules, glycogen-positive hepatocyte counts, Kupffer cell counts, and the hepatic sinusoid-to-hepatic cord area ratio using digital image analysis. Data were analyzed using one-way analysis of variance followed by Tukey's multiple-comparison test, with statistical significance established at p < 0.05.

**Results:**

Significant production system-associated differences were observed in all major histomorphometric parameters. Beta-agonist-free swine exhibited the greatest hepatic glycogen deposition (34.23 ± 2.74%) and the highest glycogen-positive hepatocyte counts (167.85 ± 12.97 cells/area), followed by natural and hygienic swine (p < 0.05). Zone-specific analysis demonstrated significantly greater glycogen accumulation across periportal, midzonal, and pericentral regions in beta-agonist-free animals. In contrast, natural swine showed significantly higher Kupffer cell abundance (3.97 ± 0.93 cells/area) and a greater hepatic sinusoid-to-hepatic cord area ratio (0.84 ± 0.14) than beta-agonist-free swine (p < 0.05), indicating enhanced hepatic immune surveillance and subtle microarchitectural remodeling. No histopathological lesions, including hepatocellular degeneration, necrosis, or inflammatory infiltration, were observed in any group.

**Conclusion:**

Different commercial swine production systems were associated with distinct hepatic histomorphometric adaptations. Beta-agonist-free production favored greater hepatic glycogen storage, whereas natural production was characterized by increased Kupffer cell abundance and expanded sinusoidal architecture, suggesting differences in hepatic metabolic status and immune microenvironment. These findings provide novel comparative evidence that husbandry practices influence liver structure in commercial swine and establish baseline histological data that may support future investigations into animal welfare, sustainable livestock production, and hepatic health assessment.

## INTRODUCTION

Pig farming systems differ substantially in their management approaches, ranging from tightly controlled indoor production units to outdoor natural environments [1, 2]. These systems vary in terms of housing conditions, sanitation practices, feeding strategies, and exposure to environmental microorganisms, all of which may influence animal physiology and metabolic status [3, 4]. In Thailand, the increasing demand for pork products produced under safer and more welfare-conscious conditions has stimulated the development of alternative production systems, including beta-agonist-free and free-range farming [5, 6]. Despite their growing importance, information regarding the biological effects of these production systems on internal organ structure and function remains limited. Swine are also recognized as valuable animal models for veterinary and biomedical research because of their physiological and anatomical similarities to humans [7, 8]. Furthermore, recent advances in artificial intelligence (AI) and digital pathology have increased the demand for reliable histological reference datasets, highlighting the continuing importance of conventional histological investigations for both veterinary science and comparative biomedical research [9, 10]. The liver plays a central role in maintaining metabolic homeostasis in mammals. It is responsible for regulating carbohydrate metabolism, storing glycogen, detoxifying metabolites, and coordinating immune-related processes within the body [11, 12]. Hepatic glycogen represents a major energy reserve and its distribution within hepatocytes reflects the metabolic balance between glucose storage and utilization [13, 14]. Variations in glycogen deposition may occur in response to nutritional status, environmental stress, or physiological adaptation [15, 16]. Histochemically, glycogen can be visualized using periodic acid–Schiff (PAS) staining, which detects polysaccharides and glycoproteins within tissues [17, 18]. PAS-positive reactions appear as magenta staining in hepatocyte cytoplasm, allowing the assessment of glycogen accumulation patterns across hepatic lobules [19, 20]. In addition to glycogen storage, structural components of the liver such as Kupffer cells, hepatic sinusoids, and hepatic cords contribute to immune defense, microcirculatory regulation, and overall hepatic function [21–23]. Evidence from previous investigations suggests that animal management conditions can influence tissue architecture in swine. Studies examining gastrointestinal tissues have reported variations in mucosal organization and secretory cell distribution associated with different farming environments [24–26].

Likewise, recent histomorphometric analyses of swine lungs demonstrated differences in pulmonary structure, fibrosis, and macrophage distribution among animals raised under distinct production systems, indicating that husbandry conditions may affect organ morphology beyond the digestive tract [27]. However, information regarding production system-associated changes in hepatic glycogen deposition, Kupffer cell abundance, and hepatic microarchitecture remains limited. Because the liver integrates metabolic and immune-related responses to nutritional, environmental, and physiological challenges, evaluation of these parameters may provide valuable insight into hepatic adaptation under different farming conditions.

Based on differences in management practices, environmental exposure, and physiological demands among production systems, we hypothesized that swine raised under different systems would exhibit distinct hepatic histomorphometric characteristics. Specifically, we expected variations in hepatic glycogen deposition, Kupffer cell abundance, and sinusoid–hepatic cord architecture, reflecting differences in hepatic metabolic and immune-related adaptation. Therefore, this study aimed to provide an integrated histomorphometric evaluation of hepatic glycogen storage, Kupffer cell abundance, and hepatic microarchitecture in swine raised under beta-agonist-free, hygienic, and natural production systems in Thailand. To our knowledge, this study is among the first to simultaneously compare these hepatic metabolic, immune-related, and structural parameters across commercially relevant swine production systems within the same geographic region of Southeast Asia. By integrating assessments of hepatic glycogen deposition, Kupffer cell abundance, and hepatic microarchitecture, this study provides a comprehensive histomorphometric evaluation of production system-associated hepatic adaptations in commercial swine.

## MATERIALS AND METHODS

### Ethical approval

Ethical approval for this study was obtained from the Animal Ethics Committee of the Center for Animal Research, Naresuan University, Thailand (Approval No. NU-AEE620505). All procedures were conducted in accordance with the institutional guidelines for animal care and use and complied with the principles outlined in the Guide for the Care and Use of Laboratory Animals published by the National Institutes of Health. Liver samples used in this study were collected post-mortem from clinically healthy market weight swine that had been slaughtered for routine commercial meat production. No animals were euthanized or subjected to additional experimental procedures specifically for the purpose of this study. Sample collection was performed after slaughter in accordance with standard hygienic practices at the processing facility, ensuring that the study did not introduce any additional handling, intervention, or distress to the animals. The design, conduct, and reporting of this study followed the recommendations of the ARRIVE guidelines 2.0 (Animal Research: Reporting of *In Vivo* Experiments) to promote transparency, reproducibility, and comprehensive reporting of animal research. Relevant ARRIVE elements addressed in this study include ethical approval, study design, sample size, animal source, housing and management conditions, and statistical analysis.

### Study period and location

This study was conducted in Thailand. Sample collection and processing were performed between July, 2025 to March, 2026 at facilities associated with Naresuan University and commercial swine farms in the region.

### Study design

This cross-sectional comparative histological study included 30 clinically healthy, market weight swine raised under three different management systems in Thailand: beta-agonist-free, hygienic, and natural production systems (n = 10 animals per group).

### Animal selection and grouping

This cross-sectional comparative histological study included 30 clinically healthy, market weight swine raised under three different management systems in Thailand: beta-agonist-free, hygienic, and natural production systems (n = 10 animals per group). Animals were obtained from representative commercial farms operating under standard management practices specific to each system within the same geographic region to minimize environmental and climatic variability. The animals consisted of Thai native × Landrace × Large White swine, including mixed sexes, with an age range of approximately 4 months and a body weight range of 100–120 kilograms at slaughter. Animals were selected based on predefined inclusion criteria, including normal growth performance, normal feeding behavior, satisfactory body condition, absence of clinical signs of disease, and no documented history of systemic illness during the production period. Prior to slaughter, health status was assessed through routine veterinary examination, including evaluation of general appearance, locomotion, respiratory function, feeding activity, and signs of gastrointestinal, dermatological, or systemic disorders. Animals exhibiting evidence of illness, injury, abnormal behavior, poor body condition, or other health abnormalities were excluded from the study. No blood biochemical or hematological analyses were performed as part of the selection process. All animals were raised under their respective production systems from the post-weaning period until slaughter to ensure physiological adaptation to the associated husbandry conditions. No experimental treatments or interventions were applied before sample collection, and liver tissues were obtained postmortem from animals slaughtered for routine commercial meat production.

### Housing and management conditions

Swine in the three management systems were maintained under distinct housing and husbandry conditions according to the standard practices of each production system. The beta-agonist-free production system followed conventional commercial intensive management practices without the administration of beta-adrenergic agonist growth promoters during the production period. Pigs were housed in semi-controlled pens with natural ventilation and routine sanitation practices. Animals received commercial feed formulated to meet standard nutritional requirements for growing–finishing swine and had *ad libitum* access to clean drinking water. The hygienic production system was characterized by enhanced biosecurity and sanitation measures, including controlled farm access, routine cleaning and disinfection procedures, and management practices intended to reduce pathogen exposure. Environmental parameters such as temperature and ventilation were maintained within recommended ranges for commercial swine production. Animals were fed standardized commercial diets and had unrestricted access to drinking water. The natural production system involved free-range or semi-free-range management with reduced confinement and increased environmental exposure. Animals were maintained according to the routine feeding and management practices of their respective farms. Although all farms provided nutritionally adequate diets appropriate for the growth stage of the animals, detailed feed formulations, nutrient composition analyses, metabolizable energy values, and individual feed intake records were not available for all production systems. Likewise, quantitative environmental measurements such as stocking density, temperature, humidity, ammonia concentrations, and pathogen monitoring data were not available. However, all farms operated under established commercial management programs representative of their respective production systems. To minimize potential confounding factors, animals included in the study were of comparable market age and body weight at slaughter and originated from farms located within the same geographic region. These measures were implemented to reduce variation associated with age, climate, and regional management differences.

### Tissue collection and sample preparation

Swine from each management system were humanely euthanized following standard veterinary procedures approved by the institutional animal care guidelines. Liver tissues were collected immediately after necropsy to minimize postmortem changes. To reduce variability associated with regional differences in hepatic architecture, liver specimens were consistently sampled from the right medial lobe in all animals. The collected tissues were gently rinsed with phosphate-buffered saline (pH 7.4) to remove residual blood and debris and were subsequently fixed in 10% neutral buffered formalin for at least 48 h to preserve tissue morphology and cellular integrity. All tissue samples were placed in fixative within 1 h of slaughter. To minimize observer bias, samples were assigned anonymized coded identifiers before histological processing and analysis, and investigators performing histomorphometric evaluations were blinded to the production system allocation of each specimen.

### Tissue processing and histological staining

Following collection and fixation, liver tissues were washed in running tap water, dehydrated through a graded series of ethanol solutions (70%, 80%, 90%, 95%, and absolute ethanol), cleared in xylene, and embedded in paraffin wax using a standard histological tissue processing protocol. Paraffin-embedded liver blocks were sectioned at 3 µm thickness using a rotary microtome, and the sections were mounted onto clean glass slides. For the visualization of glycogen and other carbohydrate-rich structures, tissue sections were stained using the PAS staining technique. Briefly, sections were deparaffinized in xylene and rehydrated through descending concentrations of ethanol to distilled water. The slides were then oxidized in periodic acid solution to generate aldehyde groups from carbohydrate components, followed by treatment with Schiff reagent, which reacts with aldehydes to produce a magenta coloration indicating PAS-positive substances such as glycogen. After staining, sections were rinsed in running tap water, counterstained with hematoxylin to visualize nuclei, dehydrated through graded ethanol, cleared in xylene, and mounted with a permanent mounting medium. PAS staining was used to evaluate relative glycogen-rich material within hepatic tissue according to established histological protocols. However, diastase digestion controls (PAS-D) were not performed to confirm glycogen specificity, and dedicated positive and negative PAS control tissues were not included in the staining procedure. Therefore, the results should be interpreted as comparative assessments of PAS-positive material among the experimental groups. All stained sections were examined under a light microscope, and representative digital micrographs were captured for subsequent histomorphometric analysis. Kupffer cells were identified based on their characteristic morphology and anatomical location along the hepatic sinusoidal lining, consistent with conventional histological assessment methods. No immunohistochemical markers, such as CD68 or Iba1, were employed for Kupffer cell identification. Histomorphometric analyses included assessment of PAS-positive glycogen area, zonal glycogen distribution within hepatic lobules (Zones 1–3), glycogen-positive hepatocyte number, Kupffer cell count, and the hepatic sinusoid-to-hepatic cord area ratio.

### Histomorphometric analysis

Histomorphometric evaluation of liver sections was performed using light microscopy coupled with digital image analysis. PAS-stained sections were examined under a light microscope using 4× to 40× objectives, and representative micrographs were captured with a calibrated digital camera. Quantitative measurements were conducted using ImageJ software (National Institutes of Health, Bethesda, MD, USA) to ensure standardized and reproducible analysis across samples. Color thresholding was performed using the ImageJ Color Threshold function in the hue, saturation, and brightness (HSB) color space. Threshold parameters were established using representative sections and then applied uniformly to all images analyzed within the study. Images were calibrated using the microscope scale bar prior to quantitative measurements. Five histomorphometric parameters were evaluated. First, the PAS-positive glycogen area was quantified by measuring the proportion of magenta-stained regions within the hepatic parenchyma relative to the total tissue area analyzed. Color thresholding was applied to selectively detect PAS-positive staining, and the percentage area of glycogen accumulation was calculated for each field. Second, the zonal distribution of glycogen within the hepatic lobule was assessed according to the classical hepatic acinar model. Zone 1 (periportal) was defined as the hepatocyte region adjacent to portal triads, Zone 2 (midzonal) as the intermediate region between portal triads and central veins, and Zone 3 (pericentral) as the hepatocyte region surrounding central veins. The predominant zone of glycogen deposition was determined for each specimen based on the relative intensity and spatial distribution of PAS-positive staining. Third, the number of glycogen-positive hepatocytes was determined by counting hepatocytes exhibiting distinct PAS-positive cytoplasmic staining within defined microscopic fields. The results were expressed as the mean number of PAS-positive hepatocytes per field. Fourth, Kupffer cell abundance was evaluated by identifying these cells within hepatic sinusoids based on their characteristic morphology and anatomical location. Cells were classified as Kupffer cells when they exhibited an irregular or stellate appearance, a prominent nucleus, and localization along the sinusoidal lining. No immunohistochemical markers, such as CD68 or Iba1, were employed for cell identification. Therefore, Kupffer cell identification was based on conventional histomorphological criteria. Finally, the hepatic sinusoid-to-hepatic cord area ratio was calculated to assess the relative spatial organization of hepatic parenchyma. Using ImageJ, the areas occupied by hepatic sinusoids and hepatocyte cords were delineated and measured, and the ratio was calculated by dividing the sinusoidal area by the hepatic cord area.

To minimize measurement bias, histomorphometric assessments were independently performed by three blinded observers using anonymized and coded images. The final value for each parameter was calculated as the mean of the three evaluations. For each animal, measurements were obtained from five non-overlapping microscopic fields selected from comparable liver regions, and the mean value was used for statistical analysis. This integrated histomorphometric approach simultaneously assessed hepatic glycogen deposition, zonal glycogen distribution, glycogen-positive hepatocytes, Kupffer cell abundance, and the hepatic sinusoid-to-hepatic cord area ratio, providing a comprehensive evaluation of hepatic metabolic, immune-related, and microarchitectural characteristics associated with different swine production systems. Formal inter-observer and intra-observer reproducibility analyses were not performed.

### Statistical analysis

All quantitative data were expressed as mean ± standard error of the mean (SEM). Statistical analyses were performed using GraphPad Prism version 17.0 (GraphPad Software, San Diego, CA, USA). Comparisons among the three swine production systems (beta-agonist-free, hygienic, and natural) were conducted using one-way analysis of variance (ANOVA), followed by Tukey’s multiple-comparison post hoc test for pairwise group comparisons. Variables analyzed included PAS-positive area, zone-specific glycogen distribution (Zones 1–3), glycogen-positive hepatocyte count, Kupffer cell count, and the hepatic sinusoid-to-hepatic cord area ratio. Prior to analysis, data normality and homogeneity of variance were assessed using the Shapiro–Wilk and Levene’s tests, respectively. All datasets met the assumptions required for parametric analysis. The individual animal was considered the experimental unit. For each animal, measurements obtained from five non-overlapping microscopic fields were averaged to generate a single representative value for each parameter, thereby avoiding pseudoreplication and ensuring independence of observations. To complement significance testing, effect sizes were calculated using eta-squared (η²) for ANOVA comparisons, and 95% confidence intervals (CIs) were reported where applicable. Potential outliers were evaluated using Grubbs’ test; however, no values met the criteria for exclusion, and all observations were retained in the final analyses. Statistical significance was established at p < 0.05, and exact p-values were reported whenever possible.

## RESULTS

### General hepatic histological architecture

**Figure 1:**
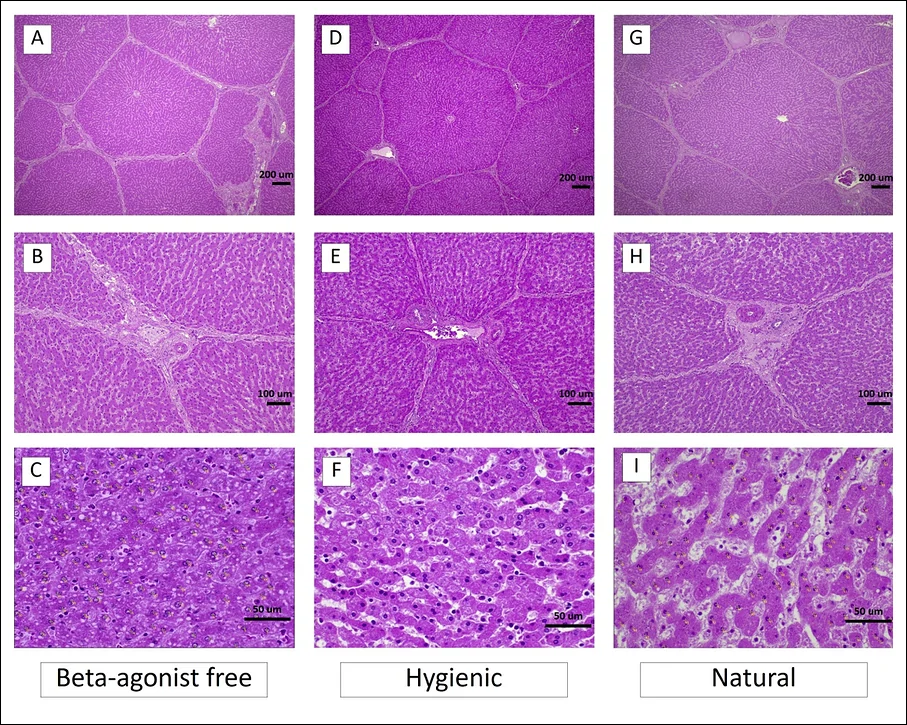
Representative hepatic histology of swine raised under different production systems. Periodic acid–Schiff (PAS)-stained liver sections from beta-agonist-free (A-C), hygienic (D-F), and natural (G-I) swine. Images were obtained at low (A, D, G), intermediate (B, E, H), and high (C, F, I) magnification. The liver parenchyma exhibited preserved hepatic lobular architecture in all groups, characterized by hepatocyte cords radiating from the central vein and separated by hepatic sinusoids. PAS-positive glycogen deposits were observed within the cytoplasm of hepatocytes, whereas Kupffer cells were identified along the sinusoidal lining. Portal triads, consisting of branches of the portal vein, hepatic artery, and bile duct, were evident at the lobular periphery. High-magnification images (C, F, I) illustrate hepatocyte morphology, PAS-positive glycogen accumulation, and Kupffer cells within hepatic sinusoids. Representative images are shown from each production system (n = 10 animals/group). Scale bars = 200 μm (A, D, G), 100 μm (B, E, H), and 50 μm (C, F, I).

Histological examination of liver sections stained with periodic acid–Schiff (PAS) revealed that the fundamental hepatic architecture was preserved in swine raised under all three management systems (beta-agonist-free, hygienic, and natural) (Figure 1). The liver parenchyma exhibited the typical lobular organization characterized by hepatocyte plates arranged radially around the central vein and separated by hepatic sinusoids. Hepatocytes appeared polygonal in shape with centrally located nuclei, and the sinusoidal spaces were lined by endothelial cells and scattered Kupffer cells. Portal triads consisting of branches of the portal vein, hepatic artery, and bile duct were also observed at the periphery of hepatic lobules. PAS staining demonstrated varying intensities of magenta coloration within the hepatocyte cytoplasm, indicating the presence of glycogen deposits. In general, PAS-positive material appeared as fine to coarse cytoplasmic granules within hepatocytes, with distribution patterns differing slightly among the three management systems. The hepatic lobular zones including Zone 1 (periportal), Zone 2 (midzonal), and Zone 3 (pericentral) could be distinguished based on their proximity to portal triads and central veins, enabling assessment of glycogen distribution across the metabolic gradient of the hepatic lobule. Despite differences in PAS staining intensity, the overall structural integrity of the hepatic cords and sinusoidal network remained comparable among the three groups. No obvious pathological alterations such as hepatocellular degeneration, necrosis, or inflammatory infiltration were observed in any of the examined sections, suggesting that the animals were clinically healthy and that the observed differences were primarily related to physiological variation in glycogen accumulation rather than hepatic pathology.

### Quantitative analysis of hepatic PAS-positive area

**Figure 2:**
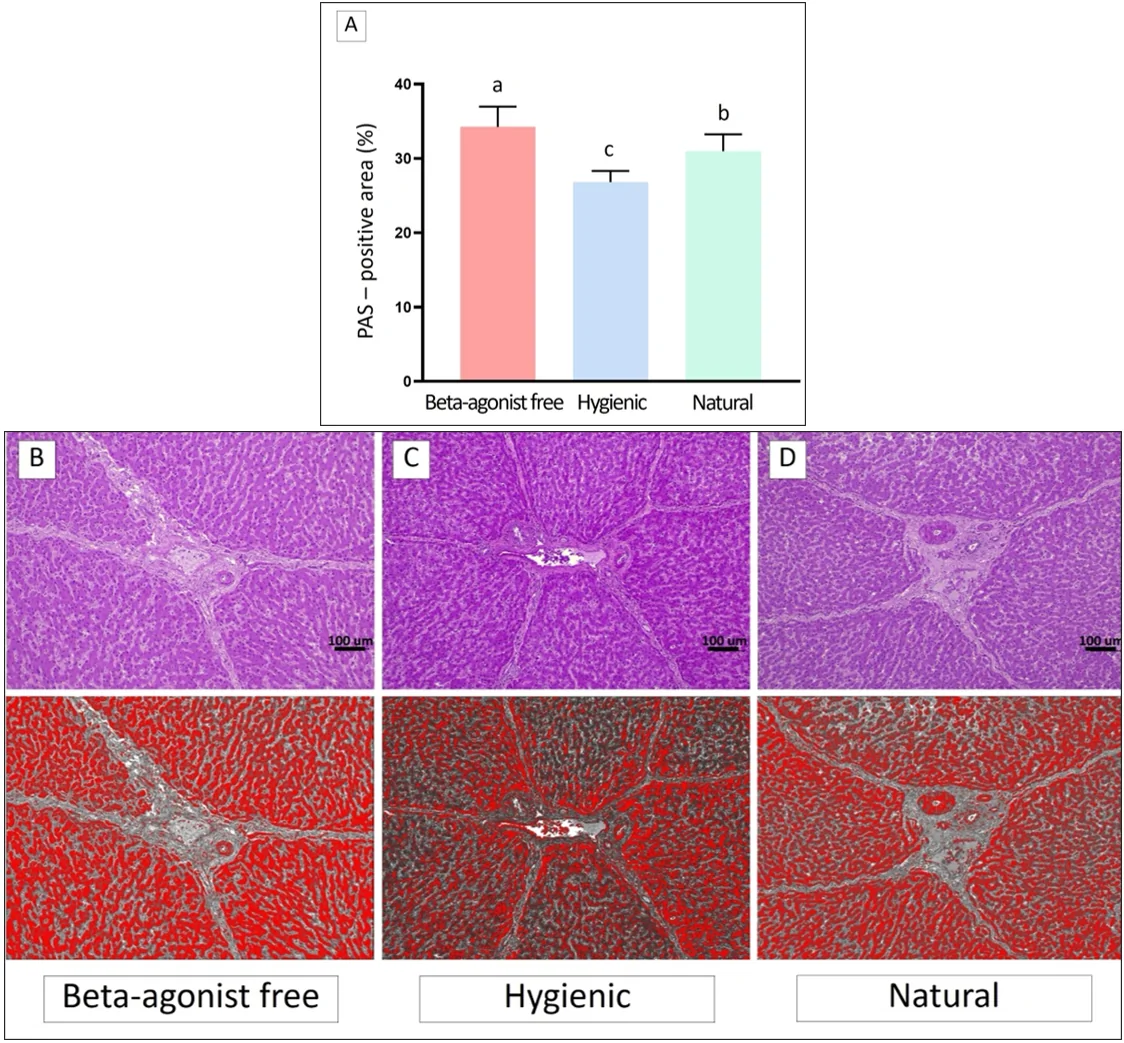
Periodic acid–Schiff (PAS)-positive glycogen distribution in the liver of swine raised under different production systems. (A) Quantitative comparison of the PAS-positive glycogen area within hepatic lobules of beta-agonist-free, hygienic, and natural swine. Data are presented as mean ± SEM (n = 10 animals/group). Bars with different lowercase letters differ significantly (Tukey's multiple-comparison test, p < 0.05). (B-D) Representative PAS-stained liver sections from beta-agonist-free (B), hygienic (C), and natural (D) swine. The upper panels show the original PAS-stained sections illustrating glycogen accumulation within hepatocyte cytoplasm, whereas the lower panels show the corresponding ImageJ-generated positive area analyses used for quantitative assessment. PAS-positive glycogen deposits appear as magenta granules distributed throughout hepatocytes within hepatic lobules. Scale bar = 100 μm.

Quantitative analysis of PAS staining demonstrated significant differences in hepatic glycogen accumulation among swine raised under different management systems (Figure 2). The proportion of PAS-positive areas within hepatic lobules was highest in the beta-agonist-free group (34.23 ± 2.74%; 95% CI: 28.03–40.43%), followed by the natural group (30.97 ± 2.28%; 95% CI: 25.81–36.13%), whereas the hygienic group exhibited the lowest value (26.80 ± 1.51%; 95% CI: 23.38–30.22%). Statistical analysis showed that the PAS-positive area in the beta-agonist-free group was significantly greater than that in the hygienic and natural groups (p < 0.0001 and p = 0.0035, respectively). In addition, the natural group demonstrated a significantly higher PAS-positive area compared with the hygienic group (p < 0.0001). The magnitude of the management system effect was large (η² = 0.171), indicating a substantial influence of production system on hepatic glycogen deposition.

### Zone-specific distribution of PAS-positive glycogen within hepatic lobules

**Figure 3:**
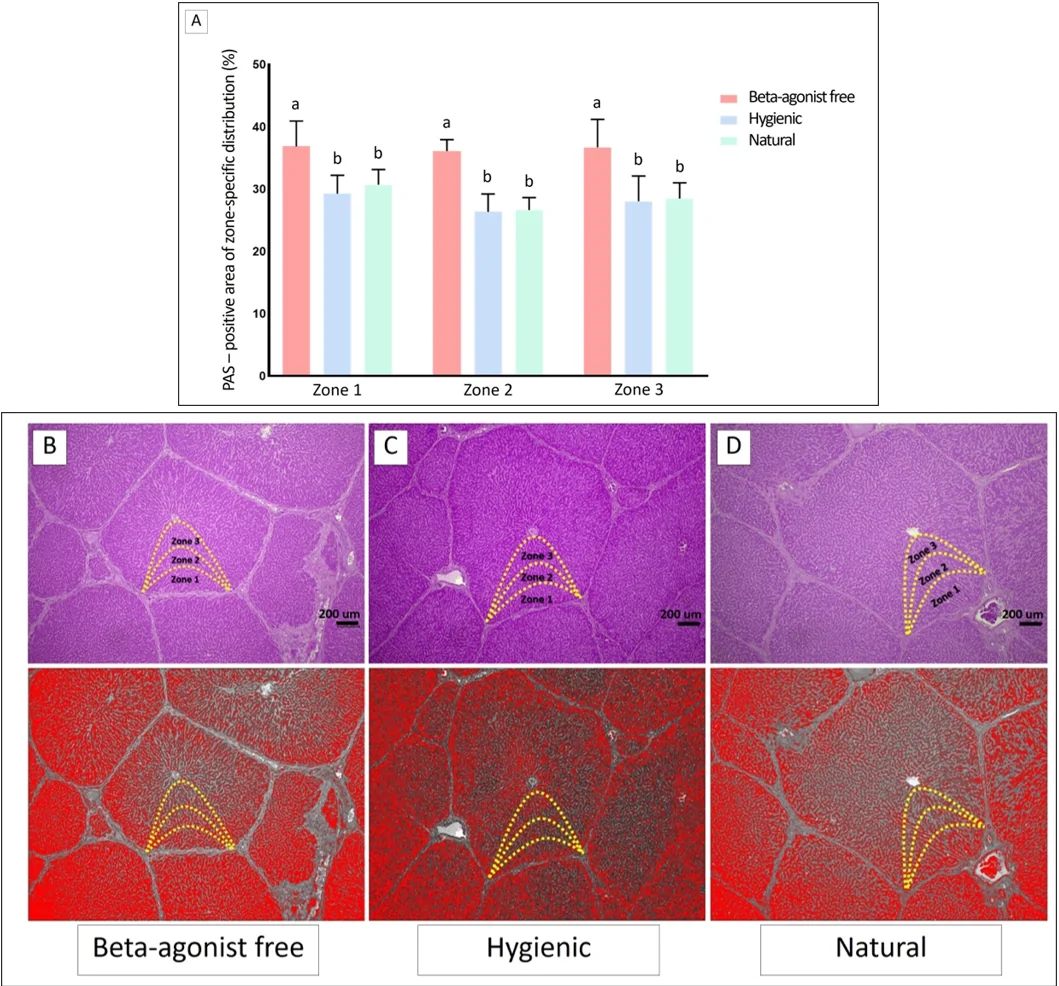
Zone-specific distribution of hepatic glycogen in swine raised under different production systems. (A) Quantitative analysis of the Periodic acid–Schiff (PAS)-positive glycogen area within hepatic lobule zones, including Zone 1 (periportal), Zone 2 (midzonal), and Zone 3 (pericentral), in beta-agonist-free, hygienic, and natural swine. Data are presented as mean ± SEM (n = 10 animals/group). Bars with different lowercase letters differ significantly (Tukey's multiple-comparison test, p < 0.05). (B-D) Representative PAS-stained liver sections from beta-agonist-free (B), hygienic (C), and natural (D) swine illustrating the zonal distribution of glycogen within hepatic lobules. The upper panels show the original PAS-stained sections, whereas the lower panels show the corresponding ImageJ-generated positive area analyses used for quantitative assessment. PAS-positive glycogen deposits appear as magenta staining within hepatocyte cytoplasm throughout the hepatic lobule. Representative images are shown from each production system (n = 10 animals/group). Scale bar = 200 μm.

Histomorphometric analysis of PAS-positive glycogen demonstrated zone-dependent distribution patterns within hepatic lobules among swine raised under different management systems (Figure 3). In the beta-agonist-free group, the PAS-positive glycogen area was 37.43 ± 5.31% (95% CI: 25.42–49.44%) in Zone 1 (periportal), 36.33 ± 3.28% (95% CI: 28.91–43.75%) in Zone 2 (midzonal), and 37.21 ± 5.86% (95% CI: 23.96–50.46%) in Zone 3 (centrilobular). In contrast, the hygienic group exhibited lower values across all zones, with 29.60 ± 4.27% (95% CI: 19.94–39.26%) in Zone 1, 26.71 ± 4.08% (95% CI: 17.48–35.94%) in Zone 2, and 28.35 ± 5.78% (95% CI: 15.28–41.42%) in Zone 3. The natural group showed intermediate values, with 30.57 ± 4.00% (95% CI: 21.52–39.62%) in Zone 1, 26.56 ± 3.78% (95% CI: 18.01–35.11%) in Zone 2, and 28.36 ± 4.33% (95% CI: 18.57–38.15%) in Zone 3. Statistical analysis revealed significant differences in PAS-positive glycogen levels among the management systems in all three zones. The beta-agonist-free group showed significantly higher glycogen levels than both the hygienic and natural groups (p < 0.0001). The magnitude of these differences was large, with effect sizes (η²) of 0.502, 0.751, and 0.473 for Zones 1, 2, and 3, respectively. The strongest production system effect was observed in the midzonal region (Zone 2), indicating substantial variation in hepatic glycogen distribution associated with management practices.

### Glycogen-stained hepatocyte counts

**Figure 4:**
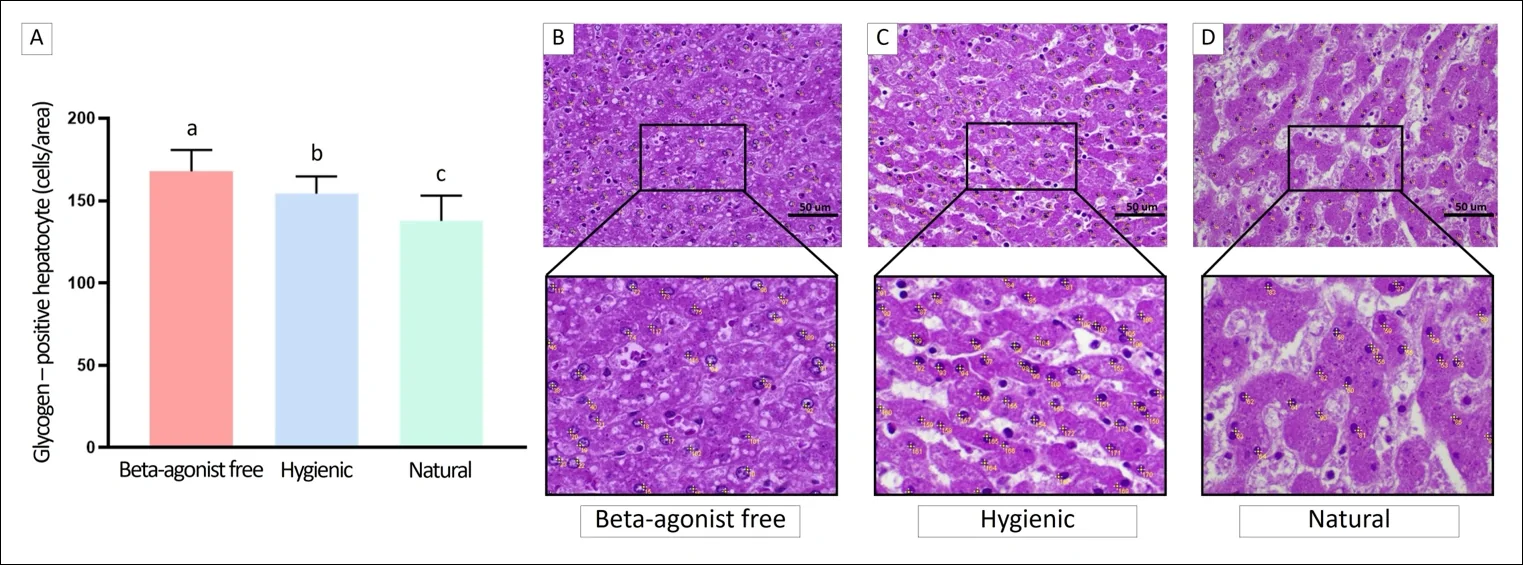
Quantitative analysis of glycogen-positive hepatocytes in the liver of swine raised under different production systems. (A) Quantitative comparison of glycogen-positive hepatocytes identified by Periodic acid–Schiff (PAS) staining among beta-agonist-free, hygienic, and natural swine. Data are presented as mean ± SEM (n = 10 animals/group). Bars with different lowercase letters differ significantly (Tukey's multiple-comparison test, p < 0.05). (B-D) Representative PAS-stained liver sections from beta-agonist-free (B), hygienic (C), and natural (D) swine. The upper panels show the original PAS-stained sections illustrating glycogen accumulation within hepatocyte cytoplasm, whereas the lower panels show enlarged views used for quantitative analysis. Glycogen-positive hepatocytes included in the quantitative analysis are indicated by yellow dots. Representative images are shown from each production system (n = 10 animals/group). Scale bar = 50 μm.

Quantitative analysis of PAS-stained liver sections revealed significant differences in the number of glycogen-positive hepatocytes among swine raised under different management systems (Figure 4). The beta-agonist-free group exhibited the highest number of glycogen-stained hepatocytes (167.85 ± 12.97 cells/area; 95% CI: 138.51–197.19), followed by the hygienic group (154.35 ± 10.48 cells/area; 95% CI: 130.65–178.05), whereas the natural group showed the lowest value (137.58 ± 15.51 cells/area; 95% CI: 102.49–172.67). Statistical analysis indicated that the number of glycogen-stained hepatocytes was significantly greater in the beta-agonist-free group than in both the hygienic and natural groups (p < 0.0001). In addition, the hygienic group demonstrated significantly higher values than the natural group (p < 0.0001). The effect of production system on glycogen-positive hepatocyte counts was moderate to large (η² = 0.090), indicating that approximately 9.0% of the observed variance was attributable to differences among management systems. 

### Kupffer cell counts

**Figure 5:**
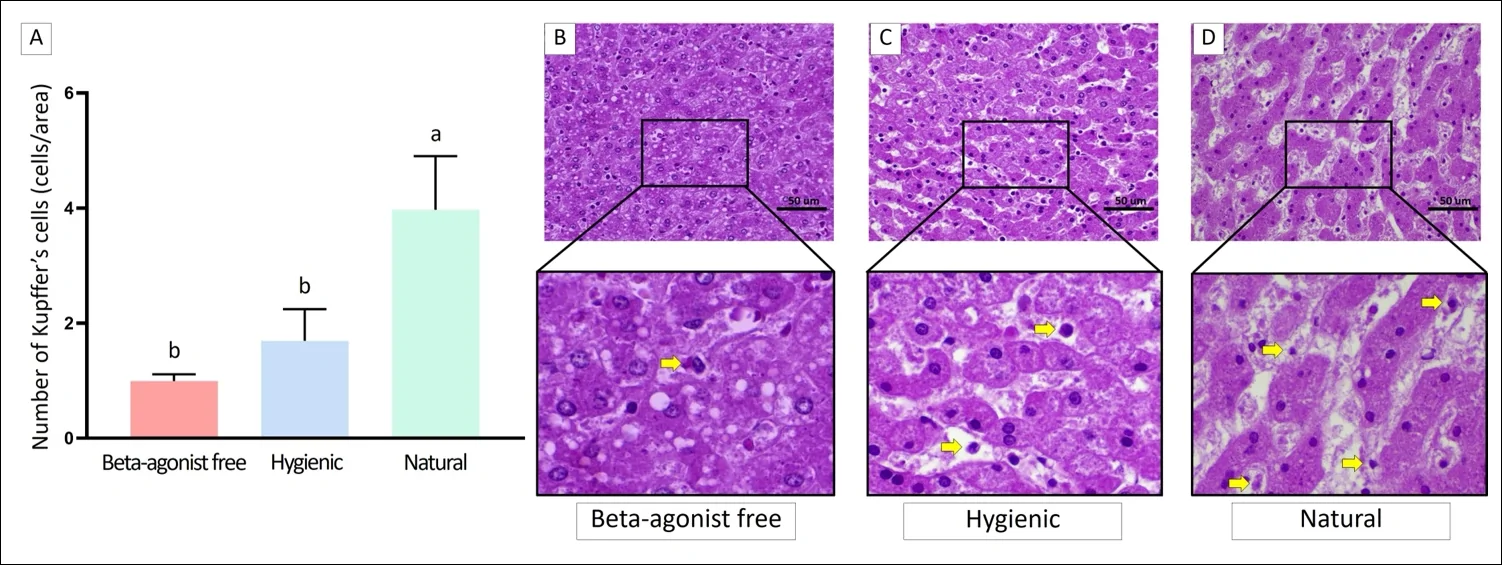
Distribution of Kupffer cells within hepatic sinusoids of swine raised under different production systems. (A) Quantitative comparison of Kupffer cell counts within hepatic sinusoids among beta-agonist-free, hygienic, and natural swine. Data are presented as mean ± SEM (n = 10 animals/group). Bars with different lowercase letters differ significantly (Tukey's multiple-comparison test, p < 0.05). (B-D) Representative liver sections from beta-agonist-free (B), hygienic (C), and natural (D) swine illustrating the distribution of Kupffer cells within hepatic sinusoids. The upper panels show the original liver sections, whereas the lower panels show enlarged views highlighting Kupffer cells located along the sinusoidal lining and identified based on their characteristic morphology. Kupffer cells included in the quantitative analysis are indicated by yellow arrows. Representative images are shown from each production system (n = 10 animals/group). Scale bar = 50 μm.

Kupffer cell distribution within hepatic sinusoids was quantitatively evaluated among the three swine management systems (Figure 5). The results demonstrated that the natural group exhibited the highest Kupffer cell count (3.97 ± 0.93 cells/area; 95% CI: 1.87–6.07), followed by the hygienic group (1.69 ± 0.55 cells/area; 95% CI: 0.45–2.93), whereas the beta-agonist-free group showed the lowest value (0.99 ± 0.12 cells/area; 95% CI: 0.72–1.26). Statistical analysis revealed a significant increase in Kupffer cell counts in the natural group compared with the beta-agonist-free group (p = 0.0028). In addition, Kupffer cell counts were significantly higher in the natural group than in the hygienic group (p = 0.0105). No statistically significant difference was observed between the beta-agonist-free and hygienic groups. These findings indicate that management systems influence hepatic macrophage distribution, with swine raised under natural conditions demonstrating a greater presence of Kupffer cells within hepatic sinusoids. The effect of production system on Kupffer cell abundance was large (η² = 0.313), indicating that approximately 31.3% of the variance in Kupffer cell counts was associated with differences among management systems.

### Hepatic sinusoid-to-hepatic cord area ratio

**Figure 6:**
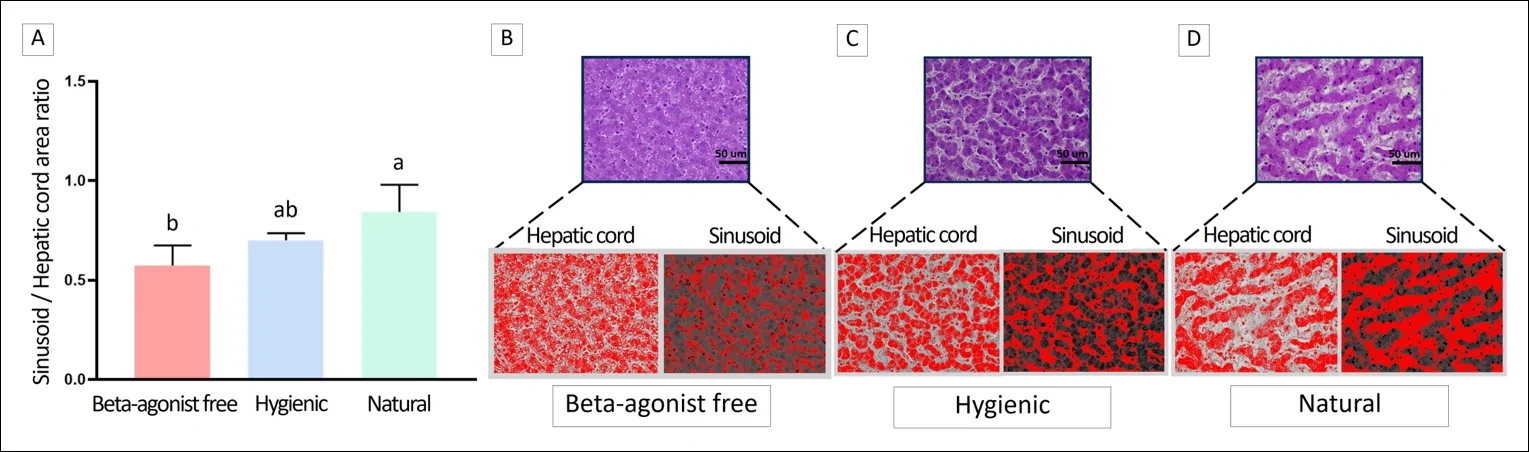
Hepatic sinusoid-to-hepatic cord area ratio in swine raised under different production systems. (A) Quantitative comparison of the hepatic sinusoid-to-hepatic cord area ratio among beta-agonist-free, hygienic, and natural swine. Data are presented as mean ± SEM (n = 10 animals/group). Bars with different lowercase letters differ significantly (Tukey's multiple-comparison test, p < 0.05). (B–D) Representative Periodic acid–Schiff (PAS)-stained liver sections from beta-agonist-free (B), hygienic (C), and natural (D) swine illustrating hepatic cords and sinusoidal spaces within hepatic lobules. The upper panels show the original PAS-stained sections, whereas the lower panels show the corresponding ImageJ-generated segmentations used for morphometric analysis. Hepatic cord areas are highlighted in red, whereas hepatic sinusoidal areas are separately delineated for quantitative measurement of the sinusoid-to-hepatic cord area ratio. Representative images are shown from each production system (n = 10 animals/group). Scale bar = 50 μm.

The hepatic sinusoid-to-hepatic cord area ratio was analyzed to evaluate structural differences in hepatic microarchitecture among swine raised under different management systems (Figure 6). The results demonstrated that the natural group exhibited the highest ratio (0.84 ± 0.14; 95% CI: 0.52–1.16), followed by the hygienic group (0.70 ± 0.04; 95% CI: 0.61–0.79), while the beta-agonist-free group showed the lowest value (0.57 ± 0.10; 95% CI: 0.34–0.80). Statistical analysis revealed a significant increase in the sinusoid–to–cord ratio in the natural group compared with the beta-agonist-free group (p = 0.0378). However, no statistically significant differences were observed between the hygienic and beta-agonist-free groups or between the hygienic and natural groups. The overall effect of management system on this parameter was small to moderate (η² = 0.054), indicating that approximately 5.4% of the observed variance in hepatic microarchitecture was attributable to differences among production systems. These findings suggest that swine raised under natural management conditions may exhibit relatively expanded sinusoidal spaces in relation to hepatic cords, reflecting subtle variations in hepatic microstructural organization associated with different production systems.

## DISCUSSION

### Overview of findings

The present study evaluated hepatic histological characteristics and glycogen distribution in swine raised under different management systems, with particular emphasis on PAS-positive glycogen accumulation, zonal distribution within hepatic lobules, hepatocyte glycogen staining, Kupffer cell abundance, and hepatic microarchitectural ratios. Overall, the findings demonstrate that swine production systems influence hepatic metabolic and structural features, particularly in relation to glycogen storage and immune cell distribution within the liver. To our knowledge, this study represents one of the first integrated histomorphometric evaluations of hepatic glycogen deposition, Kupffer cell abundance, and sinusoid–hepatic cord architecture in commercial swine raised under distinct production systems within the same geographic region of Thailand. Together with our previous investigations of production system-associated histological variation in other organ systems, these findings contribute to a broader understanding of organ-specific adaptations associated with commercial swine production practices.

### Hepatic glycogen deposition and metabolic implications

Quantitative analysis of PAS-positive areas revealed that beta-agonist-free swine exhibited the highest glycogen-associated staining, followed by the natural group, whereas the hygienic group demonstrated the lowest values. These differences suggest variations in hepatic carbohydrate storage and metabolic status among animals raised under different management conditions [28, 29]. Glycogen accumulation in hepatocytes is influenced by nutritional intake, metabolic demand, and hormonal regulation, and therefore may reflect differences in feeding regimes, physical activity, or physiological adaptation associated with each production system [20, 30]. Similar histological studies in livestock have indicated that hepatic glycogen levels can serve as an indicator of metabolic balance and nutritional status, with higher glycogen reserves generally reflecting favorable energy availability and efficient hepatic carbohydrate metabolism [31–33]. Hepatic glycogen storage is also highly responsive to endocrine regulation, particularly glucocorticoid-mediated glycogen mobilization during periods of physiological stress [34, 35]. Consequently, differences in environmental conditions, husbandry practices, and stress exposure among production systems may have contributed to the distinct glycogen accumulation patterns observed in the present study.

### Zone-specific glycogen distribution

The zonal analysis of PAS-positive glycogen within hepatic lobules further demonstrated distinct distribution patterns across the three groups, particularly within periportal regions (Zone 1). The beta-agonist-free group showed greater glycogen accumulation compared with the hygienic group, whereas the natural group exhibited intermediate or regionally variable values. Hepatic metabolic zonation is a well-recognized phenomenon in which hepatocytes within different lobular zones perform specialized metabolic functions [36–38]. Periportal hepatocytes are primarily associated with oxidative metabolism and gluconeogenesis, whereas pericentral regions are more closely involved in glycolysis and xenobiotic metabolism [39–41]. Variations in glycogen distribution across these zones may therefore reflect differences in metabolic regulation, nutrient availability, and physiological adaptation associated with different rearing environments [42, 43].

### Glycogen-positive hepatocytes

Consistent with the PAS-positive area findings, the number of glycogen-stained hepatocytes was significantly greater in beta-agonist-free swine compared with the hygienic and natural groups. These results reinforce the observation that management systems can influence hepatocellular glycogen storage at both the tissue and cellular levels. Although greater glycogen accumulation may indicate relatively higher hepatic energy reserves, the observational nature of the present study precludes conclusions regarding enhanced metabolic performance or efficiency. Reduced glycogen staining may reflect increased metabolic utilization or altered dietary energy balance [44–46]. Such histological differences have also been reported in animal studies evaluating metabolic adaptation to environmental or nutritional stressors [47–49].

### Kupffer cell abundance and immune implications

In contrast to glycogen-related parameters, the distribution of Kupffer cells showed an opposite trend, with the highest counts observed in the natural group, followed by the hygienic group, and the lowest values in beta-agonist-free swine. Kupffer cells are specialized hepatic macrophages that play critical roles in immune surveillance, pathogen clearance, and inflammatory regulation within the liver [50, 51]. The increased Kupffer cell presence observed in naturally raised swine may be associated with greater environmental microbial exposure, which could stimulate hepatic immune activity through gut–liver axis interactions [52–54]. Animals raised under natural or less controlled environmental conditions may encounter a wider range of environmental antigens, potentially contributing to increased macrophage activation within hepatic sinusoids [55–57]. Emerging evidence indicates that alterations in intestinal microbiota composition and microbial-derived metabolites can modulate hepatic immune responses and Kupffer cell activity through gut–liver communication pathways [58, 59]. Furthermore, previous studies have demonstrated that alterations in intestinal morphology and gut health can influence host physiological and immune responses, supporting the concept that changes within the gastrointestinal environment may have downstream effects on hepatic immune regulation through the gut–liver axis [60]. Such mechanisms may partially explain the greater Kupffer cell abundance observed in naturally raised swine and warrant further investigation using microbiome-based approaches.

### Hepatic microarchitecture

Structural analysis of hepatic microarchitecture further revealed that the sinusoid-to-hepatic cord area ratio was highest in the natural group, indicating relatively expanded sinusoidal spaces compared with hepatocyte cords. This morphological feature may reflect subtle adaptations in hepatic microcirculation or immune cell trafficking within the liver. Enlarged sinusoidal spaces may facilitate increased interaction between circulating immune cells and hepatic macrophages, particularly in environments with higher antigenic exposure [61–63]. In contrast, the lower sinusoid–cord ratio observed in beta-agonist-free swine may suggest more compact hepatocellular organization associated with relatively stable metabolic conditions [64, 65].

### Integrated interpretation and confounding factors

Interestingly, the production systems exhibiting greater hepatic glycogen deposition tended to show lower Kupffer cell abundance and reduced sinusoid-to-hepatic cord ratios, whereas the opposite pattern was observed in naturally raised swine. Although the biological significance of this observation remains uncertain, it may reflect differing hepatic adaptive responses to production system-specific environmental and physiological conditions. This apparent contrast between metabolic and immune-related hepatic parameters warrants further investi-gation through functional, molecular, and multivariate analytical approaches. Several potential confounding factors should be considered when interpreting the present findings. Although animals originated from the same geographic region and were of comparable age and market weight, differences in diet composition, nutrient intake, genetic background, environmental exposure, transport duration, fasting period prior to slaughter, and acute stress responses may have influenced hepatic glycogen metabolism and tissue architecture. Because these variables were not experimentally controlled, their contribution to the observed differences cannot be completely excluded.

### Clinical and practical implications

From a practical perspective, the observed differences in hepatic glycogen storage and microarchitecture may have implications for livestock management, animal welfare assessment, and production system certification programs. Hepatic glycogen reserves are linked to postmortem energy metabolism and may influence meat quality characteristics such as ultimate pH and water-holding capacity, whereas Kupffer cells play important roles in hepatic immune surveillance and pathogen clearance [66, 67]. Therefore, histological indicators of hepatic condition may provide useful complementary biomarkers for evaluating animal well-being and production system-associated physiological responses. Improved understanding of liver health in commercial swine production may contribute to the development of sustainable management strategies that balance productivity, animal welfare, food quality, and consumer expectations. These considerations are particularly relevant in Thailand, where diverse production systems coexist under tropical environmental conditions.

### Limitations and future directions

This study has several limitations. First, the cross-sectional design allows identification of associations but does not establish causal relationships between production systems and hepatic histological characteristics. Second, glycogen assessment relied on PAS staining without diastase digestion (PAS-D) confirmation, and therefore contributions from other PAS-reactive carbohydrates cannot be completely excluded. Third, Kupffer cells were identified using conventional morphological criteria without immunohistochemical confirmation using macrophage-specific markers. Fourth, formal inter-observer and intra-observer reproducibility analyses were not performed. Fifth, functional metabolic, biochemical, and molecular indicators of hepatic activity were not evaluated. Finally, all animals originated from farms within a single geographic region of Thailand, which may limit the broader generalizability of the findings. Future studies should incorporate larger sample sizes, multiple geographic regions, and longitudinal study designs to further elucidate the effects of production systems on hepatic physiology. The application of immunohistochemical techniques, molecular analyses, transcriptomic profiling, metabolic assessments, and gut microbiota characterization would provide deeper insight into the mechanisms underlying hepatic glycogen regulation and immune function. Such approaches may contribute to a more comprehensive understanding of production system-associated hepatic adaptations and support sustainable, welfare-oriented livestock production within a One Health framework.

## CONCLUSION

This study demonstrated production system-associated differences in hepatic histomorphometric characteristics in market weight swine. Beta-agonist-free swine exhibited the highest hepatic glycogen deposition (34.23 ± 2.74% PAS-positive area) and the greatest number of glycogen-positive hepatocytes, whereas natural production systems showed significantly higher Kupffer cell counts (3.97 ± 0.93 cells/area) and a higher sinusoid-to-hepatic cord area ratio (0.84 ± 0.14). These findings indicate distinct hepatic metabolic, immune, and microarchitectural adaptations linked to different management practices.

The observed hepatic adaptations highlight the influence of production systems on liver metabolism and immune status. Beta-agonist-free systems may support greater energy reserves, while natural systems appear associated with enhanced immune surveillance. These insights can inform management strategies to optimize animal health, welfare, meat quality, and sustainable pork production in Thailand and similar regions.

This study provides the first integrated histomorphometric evaluation of hepatic glycogen deposition, Kupffer cell abundance, and sinusoid–hepatic cord architecture across three commercially relevant swine production systems within the same geographic region, offering valuable comparative baseline data.

The cross-sectional design and relatively small sample size (n=10 per group) limit causal inferences. Glycogen assessment relied on PAS staining without diastase confirmation, Kupffer cells were identified morphologically without immunohistochemistry, and functional/metabolic parameters were not evaluated. Regional specificity may also restrict broader generalizability.

Future studies should incorporate larger, multi-regional cohorts, longitudinal designs, immunohisto-chemical validation, molecular/transcriptomic analyses, gut microbiota characterization, and functional metabolic assessments. Such work would deepen mechanistic understanding and support evidence-based improvements in swine production systems.

Different swine production systems induce distinct hepatic adaptations, with beta-agonist-free systems favoring glycogen storage and natural systems associated with increased Kupffer cell presence and sinusoidal expansion. These findings underscore the importance of production system-specific physiological responses and provide a foundation for further research aimed at enhancing animal welfare, metabolic efficiency, and sustainable livestock production.

## DATA AVAILABILITY

All data generated or analyzed during this study are included in this published article. The raw histological images and quantitative datasets supporting the findings of this study are available from the corresponding author upon reasonable request.

## GENERATIVE AI DECLARATION

The authors used generative artificial intelligence (AI) tools solely for grammar checking and language correction during manuscript preparation. The tools were not used to generate scientific content, analyze data, interpret results, or develop conclusions. All content was reviewed and verified by the authors, who take full responsibility for the manuscript.

## AUTHORS’ CONTRIBUTIONS

NC: Contributed to study design, data analysis, and manuscript preparation and revision. AN, NK, and SK: Conducted the laboratory experiments and collected, analyzed, and interpreted the data. PJ and IP: Contributed to the study design and data interpretation. CP: Conceived and designed the study, interpreted the data, and critically revised the manuscript. All authors have read and approved the final manuscript.
